# The Life and Labors of Laennec: An Introductory Address Delivered at the New Orleans School of Medicine, November 14, 1859

**Published:** 1859-12

**Authors:** Austin Flint

**Affiliations:** Professor of Clinical Medicine, etc.


*The Life and Labors of Laennec:* An Introductory Address delivered at the New Orleans School of Medicine, November 14, 1859.

By Austin Flint, M. D., Professor of Clinical Medicine, etc.

It is customary in our American colleges to signalize the beginning of each annual session of instruction by an address on the part of the Faculty, by one of its members. In some countries the event is made the occasion for imposing ceremonies.—It accords with our notions of republican simplicity to dispense with these, and the only formality with us which marks the opening of a new collegiate term, is the time honored "introductory," usually given on successive years, by the different members of the Faculty in rotation. The privilege of addressing those who are assembled on this, the opening day of the fourth regular course of lectures in the New Orleans School of Medicine, has fallen to my lot. As the organ of the Faculty, it is my pleasing duty to salute you in their behalf. I am commissioned to extend to you a cordial greeting. We welcome you to this city and to this institution. We have invited you hither under the belief that the relations of teachers and pupils which we are to sustain towards each other, will prove mutually agreeable and advantageous. We thank you for having accepted our invitation, and we firmly trust that the wishes and expectations, on both sides, with which we meet, may be more than fulfilled.

In view of the relations about to be assumed, the present occasion is one, not only of interest, but of moment to the Faculty, as well as to yourselves. On our part, we have undertaken to guide and assist your efforts in preparing yourselves for the practice of medicine. The amount of instruction which you will receive in the several departments of medical science, forms but a part, albeit a very important part, of this undertaking. It is not arrogating too much to say that the character and usefulness of the positions which you are hereafter to occupy in life, will be likely to be affected, in no small degree, by the thoughts, views and aims derived from your teachers in the lecture room, at the bed-side of the sick, and in social intercourse. We feel the responsibility which attaches itself to us in regard, not only of the prelections from our respective chairs, butof the influence which we cannot fail, if we would, to exert, as members of the medical profession, and as men, as well as teachers. Under a deep sense of the importance of our official duties, together with the precepts and examples inseparable from our unofficial relations, we pledge our strenuous exertions in behalf of your best interests. But the whole of our duty is not yet expressed. The character, success and prosperity of the school with which we are connected, are committed into our hands. Thence rests upon us a weighty obligation. Our position as a faculty, in this point of view, involves more than an ordinary amount of responsibility. The New Orleans School of Medicine is a youthful institution. It is about to enter on its fourth annual stadium. Thus far, it has advanced with a rapidity almost unprecedented. It is at this moment fairly entitled to rank among the most prominent and flourishing of the medical institutions of our country. Its progress is, and is to be, onward; and it is for us to secure the fulfillment of its destiny by unremitted exertions.

On your part, gentlemen, there are duties and responsibilities. You are bound to avail yourselves, to the utmost, of the educational advantages which are here at your disposal. This will require attention, diligence, industry. The opportunities which you may enjoy for acquiring knowledge in the several departments of medical science, are ample. In certain branches, viz: anatomy, surgery, midwifery and clinical medicine, the resources for instruction, which this city affords, are unequaled. The great Charity Hospital, a household word as familiar throughout the length and breadth of this land as the name of our city, offers facilities for acquiring practical knowledge not surpassed by those of any other institution on this continent. I say this with due deliberation. Where is the hospital equal to this in size and scope to which the medical student has free access at all times and where clinical teaching and pathological researches are free from all restrictions ? Is there an American medical school elsewhere than in New Orleans, in which material for instruction in anatomy, abundant, and furnished to the student gratuitously, is obtained with the sanction of law, without the need of secrecy or of formalities which render legal provisions inoperative! Thanks to the medical profession and enlightened legislators of New Orleans, for the wise and liberal policy which has made the Charity Hospital conducive to the interests of humanity, by being subservient to medical education, as well as an asylum for the sick! Honor to Louisiana, the only State in the Union, in which the legalization of the study of anatomy is complete!

The mutual relations which are to exist henceforward between the faculty of this school and the class now for the first time assembled, involve a host of subjects which might be appropriately considered on the present occasion. But it would be difficult to find a subject which has not been the theme of many discourses; nor would it be easy to select a field of remark into which you had not been lead already by your own reflections. Custom allows, and indeed necessity requires, a wide latitude as regards the topics which may enter into an introductory address. I propose to occupy the hour in a way which I hope will not be deemed inappropriate, and which will, at least, have the advantage of directing the current of our thoughts from the time-worn channels. It has occurred to me that it may be interesting and profitable to sketch the biography of some one of the illustrious men whose lives and labors have shed lustre on our profession. A great and good man lives not alone for his contemporaries, but for those who come after him. The works which he achieves are but a part of the inheritance which he leaves to posterity. He outlives, in this world, his mortal life, in the never dying influence of his goodness and greatness. In selecting biography for this occasion, I have chosen from a long list of honored names, one sufficiently familiar, but which will, perhaps, suggest to your minds when it is pronounced, nothing more than the brilliant discovery with which it is identified. I ask your attention to a brief survey of the life and labors of Laennec.

Rene Theophile Hyacinthe Laennec was a native of Lower Brittany, in France. He was born in 1781. His father was an advocate of some distinction, and a man of genius, but of erratic habits. The responsibility of the parental relation appears to have been transferred, at an early age, to a brother of his father, a physician of eminence, and a professor of medicine and materia medica, in the school at Nantes. His studies were directed by this uncle. At the age of eighteen, he had distinguished himself for his proficiency in preparatory studies, and had made sufficient progress in medicine to be appointed, temporarily, an assistant surgeon in the army. At the age of nineteen, he left the military service and went to Paris to complete his medical education. He attached himself to the service of Corvisart, at the hospital *la Charite.* He graduated as Doctor in Medicine, four years afterward, in 1804.

It would be interesting to know the thoughts, the aspirations, the experiences of such a man as Laennec, during his four years of studentship at Paris, between the ages of nineteen and twenty-four. These could only be known through his own confessions or the revelations of intimate friends, and they have not been disclosed. But we are not in the dark as regards his habits of industry, and the work which he performed during this interesting and important period of his life. During the first three years of his attendance at *la Charite*, he drew up a minute history of nearly four hundred cases of disease, and attended the medical lectures at the School of Medicine. He was distinguished among the host of students for his zeal in medical pursuits, and by his knowledge of the ancient languages. In his twenty-first year he began to publish contributions on important subjects. Several valuable papers from his pen appeared in the *Journal de Medicine*, conducted by Corvisart, Leroux and Boyer. The two chief prizes in medicine and surgery granted by the institute of France, were awarded to him during this year. He also, in the same year, published a memoir on cases of peritoneal inflammation. He subsequently wrote reviews for the *Journal de Medicine*, was the author of a paper describing the tunics investing the abdominal viscera, read a memoir before the Faculty of Medicine, on a variety of hydatids, to which he was the first to give the name of *acephalocysts*, and during the last year of his pupilage, gave a course of lectures on pathological anatomy.

Let me commend to your consideration, gentlemen, students of medicine, this student-life of this man of genius. None will deny to Laennec the quality of genius; yet, behold what an amount of labor he performed while preparing, as you now are, for the active duties of medical practice. Far be it from me to doubt that Providence sees fit to endow different minds with different degrees and kinds of talent, nor would I derogate, if I could, an iota from the transcendent abilities of Laennec, which God bestowed upon him; yet, can it be doubted, that his achievements in after years were in no small measure, due to the habits of study, the intellectual training, and the learning, which were the fruits of his zealous exertions in early life! Ah! gentlemen, be not so deceived as to suppose, on the one hand, that superior natural powers of mind can ever supersede the necessity of industry, nor, on the other hand, that the want of an inborn superiority renders industry useless. The Creator has implanted in every intelligent being, capacity to fulfil a certain mission in this world; what that mission is, human intelligence cannot foresee, and whether it be, or be not fulfilled, depends on the diligence with which the faculties are cultivated, and opportunities improved.

Having graduated at the age of twenty-four, he engaged in the practice of medicine, but without relinquishing his devotion to medical studies. He conducted, as chief editor, the *Journal de Medicine* for five years, and he continued to give lectures on pathological anatomy for two years. The duties of practice obliged him to discontinue the latter. He entertained, for a long time, the idea of preparing a work on that subject. He did not cease to prosecute pathological investigations, and he contributed various articles to the Dictionary of Medical Sciences. He discovered, and named the varieties of malignant disease, still known as encephaloid, or medullary cancer, and melanosis.

His appointment as chief physician to the Neckar Hospital, twelve years after his graduation, was an important event in his career of labor. Here it was, that, shortlv after entering on the duties of his office, he made the grand discovery with which his name will live to the end of time. He devoted two years to clinical observation, in developing the application of auscultation to diagnosis, before he published his discovery. He was not in haste to cry eureka! In June, 1818, he presented an outline of the new method, in a memoir which he read before the Academy of Sciences. The manner in which he was led to the discovery of auscultation is described by him with a simplicity and modesty so beautiful and characteristic, that I cannot forbear to quote his own language. He says: "In 1816, I was consulted by a young woman, laboring under general symptoms of diseased heart, and in whose case, percussion and the application of the hand, were of little avail, on account of the great degree of fatness. The other method just mentioned,* being rendered inadmissible, by the age and sex of the patient, I happened to recollect a simple and well known fact in acoustics, and fancied it might be turned to some use on the present occasion. The fact I allude to, is the great distinctness with which we hear the scratch of a pin at one end of piece of wood on applying our ear to the other. Immediately, on this suggestion, I rolled a quire of paper into a kind of cylinder, and applied one end of it to the region of the heart, and the other to my ear, and was not a little surprised and pleased, to find that I could thereby perceive the action of the heart in a manner much more clear and distinct, than I had ever been able to do by the immediate application of the ear. From this moment, I imagined that the circumstance might furnish means for enabling us to ascertain the character, not only of the action of the heart, but of every species of sound produced by the motion of all the thoracic viscera, and consequently, for the exploration of the respiration, the voice, the *rales*, and perhaps, even the fluctuation of fluid effused in the pleura or pericardium. With this conviction, I forthwith commenced at the hospital Neckar a series of observations from which I have been able to deduce a set of new signs of diseases of the chest, for the most part, certain, simple and prominent, and calculated, perhaps, to render the diagnosis of the diseases of the lungs, heart and pleura, as decided and circumstantial, as the indications furnished to the surgeon by the introduction of the finger, or sound in the complaints wherein these are used.

How unaffected and unassuming the tone of this narrative! And yet, two years of unbiased experimental observation had enabled Laennec to comprehend and demonstrate the vast importance of his discovery. He traces the discovery to a casual

*Viz: the direct application of the ear to the chest.

suggestion. Was it, then, merely the result of accident, the offspring of a lucky thought? We are told that Newton attributed the origin of the researches which led to the discovery of the law of gravitation to his having chanced to speculate on the falling of an apple to the ground. The human mind is fond of the marvellous, and it is often ingenious in accounting for the eminence of others on the score of good fortune. It is, therefore, apt to seize with avidity on such incidents, to which, with the humility often associated with true greatness, discoverers attach undue importance. The use of the paper cylinder was simply the circumstance which marked the birth of the idea of physical exploration in the mind of Laennec. Applying the ear to the chest was not new. It was practiced even by Hipocrates. Laennec had been accustomed to listen to the sounds of the heart, by the direct application of the ear to the walls of the chest, and he would have resorted to this procedure in the case of the young woman referred to in his narrative, were it not for certain reasons which he assigns. Moreover, the impromptu stethoscope, except in that particular instance, offered no advantages over the direct application of the ear. The occasion was memorable, not for the origin of the stethoscope, but as the point of departure for that "series of observations," from which the gifted mind of Laennec, with industry and perseverance, was "able to deduce a set of new signs of diseases of the chest." It may seem to have been accidental that his mind was led into this field of research. But events of such importance are not left to chance. Providence ordained the circumstance which awakened and directed the spirit of inquiry; and the genius of Laennec is manifested in the fruits of patient, skillful investigation. So it is with the so-called favored few, to whom the world is indebted for great discoveries and improvements. It is not because the opportunity has been offered to them alone, that they have fulfilled their mission, but they have had the sagacity to perceive, the spirit to strive, the boldness to execute, and last, not least, that inestimable quality —patient industry.

The memoir ef Laennec communicated to the Academy of sciences, was received with approbation by that distinguished body. A committee of three, to whom it was referred, reported in complimentary terms, and the report was adopted by the academy. The discovery did not provoke, to much extent, opposition and ridicule, springing from professional jealousy. This is probably to be explained, in a great measure, by the universal and unlimited confidence felt in the moral, as well as intellectual character of the discoverer, and the singular modesty with which the discovery was made known. It is amusing, however, at this day, when the full value of auscultation is understood, to refer to the report of the committee of the Academy of sciences, on the subject, in 1818. The report occupies three printed octavo pages, and is signed by Portal, Pelletan and Percy, names now comparatively unknown. The committee give their solemn testimony, that the sound of respiration may be heard over the healthy chest, and that they were able to distinctly hear the sounds of the heart through the stethoscope ; and they conclude that the operation of the discoverer, with regard to the possibility of obtaining, by means of auscultation, certain signs of different maladies, affecting the heart and lungs, is probably correct ! Lest they might be led to accord too much to Laennec, they were careful to state that the idea of applying the ear to the chest, was not new, since Hippocrates suggested this procedure in order to ascertain the existence of empyema ; and that physicians had been for a long time accustomed to apply the ear over the præcordial region to determine the force of the beating of the heart.

The cautious reserve evinced in the report is not to be wondered at. The vast importance of the subject, and the influence which it was destined to exert on practical medicine, could not have been foreseen. Laennec, himself, confesses that auscultation, as developed by his subsequent labors, far surpassed the estimate which he placed upon it in the early part of his researches. It is a curious feature of the report, as it is of the memoir and subsequent publications of Laennec, that the essence of the discovery was supposed to consist in the application of the wooden cylinder, or the original stethoscope. This instrument is described by the committee with minuteness, and they state, on the verbal authority of Laennec, that no definite signs of disease are to be obtained by the application of the ear directly to the chest. It does not derogate from the merit of Laennec, as a discoverer, nor from the value of his researches, that the stethoscope has been proved to be by no means essential to the practice of physical exploration; that the ground on which he based the application of this instrument, viz: a supposed power of intensifying transmitted sound, is an error in physics, and that all the signs of cardiac and pulmonary diseases, might have been discovered by the ear applied immediately to the chest. Laennec's claims to immortality rest on the opening of a new field for exploration, and on the developments resulting from his own labors in this field, and not on the employment of the wooden cylinder, which subsequent experience has shown to be unnecessary, and which late improvements have rendered comparatively useless-

Other countries were not remarkably tardy in recognizing the importance of auscultation. Sufficient delay to test, by means of clinical observations and autopsical examinations, the correctness of the principles of the new science, was but natural and proper. But despite wellknown national prejudices, it was speedily transported across the English channel and found favor with the British observers. Some of the most eminent of these soon became its most zealous expounders and contributed important additions to the facts developed by Laennec. The names of Stokes, Elliotson, Williams, Hope and Fuller are to be especially mentioned in this connection. The latter was made particularly instrumental in the diffusion of knowledge on the subject, by translating the writings of Laennec.

In our own country, within ten years from the date of Laennec's memoir to the academy of sciences, auscultation was zealously studied and advocated. One of the earliest American disciples of Laennec who was active in the importation of the doctrines of his master, was the late Dr. John D. Fisher, of Boston. As early as 1832, when I attended medical lectures in Boston, James Jackson, the professor of medicine, then about to retire from medical teaching, but who still lives, an honored practitioner, and a venerable student in medicine, was earnestly engaged in the subject of physical exploration. He never failed to carry the stethoscope durfng his hospital visits, and the signs of cardiac and pulmonary diseases entered largely into his clinical instructions. At that time his son, James Jackson, Jr., whose ultimate death, at the threshold of a medical career full of promise, is ever to be deplored, was enthusiastically prosecuting the same studies in Paris, under the guidance of the eminent Louis, and had already opened up a branch of inquiry with respect to the signs pertaining to the act of expiration, which will ever identify his name with the subject of auscultation. Since I have been led incidentally into this train of remark, it would not be less unjust than unpatriotic, were I to omit to mention the names of Oliver Wendell Holmes, John B. S. Jackson, Samuel Jackson, Gerhard and Sweet as those who have contributed greatly toward the progress in this country of the study of the physical exploration of the chest.

The first edition of the great work of Laennec on auscultation, was published after a little more than a year from the date of his memoir communicating his discovery to the academy of Sciences. This work was entitled, *Mediate Auscultation, or a treatise on the diagnosis of disease of the Lungs and Heart, based chiefly on the new method of exploration.* In this treatise the knowledge to be obtained by auscultation is presented with a completeness truly astonishing. It is in this fact that the intellectual greatness of Laennec is most apparent. It has been justly said that there is perhaps no other instance of a discovery of equal importance being brought to such perfection by the hands of the discoverer. I do not design, on this occasion, to discuss the subject of the physical signs as developed by the labors of Laennec. It would be impossible to do this within the limits of a single discourse, and it belongs to the course of didactic instruction upon which we are about to enter. Suffice it to say here that, although during the forty years which have elapsed since the publication of Laennec's works, the application of physical exploration has been considerably extended, and rendered more complete in many of its details, the fundamental truths presented by the discoverer of auscultation, not only remain as the basis of the new science, but form a large portion of the existing superstructure. Let the student become familiar with all that is now known on this subject, and he will then read the writings of Laennec with amazement that there remained so little to be altered or added.

The unremitting devotion, for more than three years, to rerearches, prosecuted in the wards of the Neckar hospital, and the dead house, together with the duties of medical practice, and the composition of his work, proved too much for a constitution never vigorous. .Shattered health obliged him to leave Paris and return to his native province in the country. It is painful to be told, as we are, by one of his biographers, that in the full tide of the success of his great discovery, when the value of his labors were acknowledged, and his position was such as should satisfy the highest worldly ambition, he was afflicted with mental despondency to such an extent as to render him weary of life. This apparent incongruity is intelligible to those who have experienced the effects of unduly prolonged and excessive mental application. Cessation of intellectual labor, and the field sports, in a short time led to restored health and energy. He foresaw, however, the consequences of a renewal of his scientific pursuits, and it is stated by a biographer, that "the great regard which he had for his family and the powerful influence of his religious principles, had alone sufficient weight to make him leave his retreat."

He returned to Paris after an absence of two years, and again entered on his hospital duties. He now began to give clinical lectures, expounding more particularly the application of auscultation. These were largely attended by students from all nations. In the following year he was appointed professor, first, of Medicine in the College of France, and afterward, of Clinical Medicine, at the hospital *la charite*, where, nearly twenty years before, he had prosecuted his clinical studies as a medical student. He declined a higher appointment, preferring, in the language of his biographer, "that which offered him an opportunity of continuing his researches and extending the knowledge of his discoveries." Another edition of his treatise on auscultation was to be prepared. In doing this, he submittəd all the facts.to a fresh examination ; he re-modelled the entire work, and took up the treatment of the diseases which, in the first edition, he had considered only in regard to the anatomy and diagnosis. With the completion of this task ended his scientific labors. The revised treatise appeared in 1826. Before its publication, cough, febrile movement, and other symptoms pointed to serious pulmonary disease. The physical signs which he had elucidated, announced but too plainly the existence of tuberculosis. It was his lot to exemplify, in his own person, one of the affections, to the knowledge of which his studies had so largely contributed; and this is one of the many similar instances which have led to the supposition that concentration of the attention upon any particular class of diseases, predisposes to their production. As a last resort, he once more sought the renovating influences of his country residence, but without avail. He died six months after the publication of the second edition of his work, at the age of forty-five. If the occasion permitted, here would be the proper place, in the course of these remarks, to speak of the importance of the labors of Laennec as regards their influence on practical medicine. We have not time to enter into a discussion of this subject, nor is it necessary. The universal value of physical signs in the discrimination of thoracic diseases, and thereby, in the successful study and treatment of these diseases, is admitted by all who have given to them sufficient attention to appreciate their importance. Suffice it to say, that cardiac and pulmonary affections, which, prior to the researches of Laennec, were among the most obscure, are now, perhaps, better understood, than the affections belonging to any other nosological class, and that this is owing mainly to improvement in diagnosis by means of physical signs, cannot be doubted. The discovery of auscultation, as the point of departure for the development of the diagnostic character derived not only from that method, but from percussion and the other divisions of physical exploration, will ever be ranked among the most memorable of the epochs belonging to the history of medicine.

The researches of Laennec connected with auscultation, constitute the crowning glory of his scientific labors, but his claims to renown by no means rest solely on these researches. His studies in pathological anatomy were productive of important results. He was the first to describe the anatomical condition of the lungs in emphysema. It is remarked by Rokitansky that this discovery, had he done nothing more, was sufficient to render his name immortal. His clinical observations relating directly to therapeutics, were not without value. His experience in the treatment of pneumonia, for example, may be cited in proof that blood-letting is not essential to the successful management of this disease. His English translator, Sir John Forbes writing at a time when the doctrines of Broussais were much in vogue, takes exception to his therapeutical views, and imputes his non-acceptance of these doctrines to personal prejudice. Time, however, has shown that in this respect, he was wiser than his critic in trusting the seductive influence of the Broussaian theory.

Of the private and domestic history of Laennec, little has been made known to the world. In the career of a man whose scientific labors began in youth, and were continued almost unceasingly, even after a fatal illness had commenced, there was probably little beyond his daily avocations, which was open to observation; and Laennec was a man to shrink from the idea of a public exposure of his inner life. Forgetfulness of self is apparent in his writings, and in the sacrifice of health, and perhaps of life, to his ardor in the acquisition and diffusion of useful knowledge. Painfully interesting and admirable is the spectacle of such a man, calmly and humbly devoting all his powers as a farewell offering to the cause of truth and the interests of humanity! He was married but two years before his death, and died childless!

It is natural to feel an intense desire to see the faces of those whom we greatly reverence, and if this be impossible, we gaze upon their portraits with deep interest. The lithographic picture of Laennec (which our worthy Dean has caused to be suspended here, on this occasion) presents a countenance and head in keeping with the intellectual and moral character of the man. The lofty and expansive forehead denotes mental endowments of no ordinary stamp. In the expression are combined the wisdom and calmness of the philosopher, with the mildness and benevolence of the philanthropist. It would require no great skill in physiognomy to know that the original of such a portrait was gifted, not only with a high order of intellect, but with those higher qualities, which make men truth-loving, conscientious, affectionate and noble-hearted.

The great amount of labor which Laennec accomplished was performed under the disadvantage of a slender constitution. His stature was small, and "during the latter years of his life, he was attenuated in a most remarkable degree, so that it was a matter of astonishment to every stranger that he could undergo the exertions which his duties required." He was fond of hunting and domestic amusements. He had a taste for practical mechanics and was accustomed to make his own stethoscopes. Through the kindness of one of my colleagues, I am so fortunate as to have in my possession a stethoscope which belonged to Laennec, and which was probably made with his own hands. This is to me a precious relic, in addition to its value as a memento of the giver. He was a proficient in classical studies, reading Greek authors with facility, and books and discourses in the Latin tongue, writing with ease and purity. In his intercourse with patients, friends and pupils, his manners were characterized by mildness and affability. The reputation which he acquired in no wise affected the humility and kindness of heart which were the most prominent traits in his moral constitution. His benevolent disposition made him ever ready to help the poor by his professional skill, and to give to this class precedence over those who were able to purchase his services. He was equally ready to assist the needy from his purse, and his private charities, unknown during his life, were found after his death, to have been unstinted. To crown all, he was a moral and a religious man. His life affords an instance, among many others, disproving the vulgar error that the pursuits of science are unfavorable to religious faith. He lived and died a firm believer in the truths of Christianity. To quote the language of one of his biographers, and his friend from youth, the distinguished Bayle, "his death was that of a Christian, supported by the hope of a better life, prepared by the constant practice of virtue. He saw his end approach with composure and resignation. His religious principles imbibed with his earliest knowledge, were strengthened by the conviction of his maturer reason. He took no pains to conceal them when they were disadvantageous to his worldly interests, and he made no boast of them when their avowal might have contributed to favor and advancement."

I have thus, gentlemen, sketched very imperfectly the life and labors of a truly great and good man. The annals of medicine furnish many such. My predilections for the studies founded by Laennec, have naturally led me to hold his memory in peculiar veneration; but it would be easy to select the names of others, whose biographies, in like manner, are redolent of greatness and goodness. The medical profession, as it seems to me, has been remiss in often failing to place on record, for the benefit of posterity, the life-histories of its distinguished members. The profession is too indifferent on the subject, and in this it does injustice to itself, as well as to the memories of those who have deserved to live in honorable remembrance. The contemplation of the lives of worthy brethren who have gone before us, is well calculated to stimulate our zeal, guide our aims, encourage us to persevere, and increase the attachment to our calling. The profession is remiss in not doing more toward popularizing knowledge of medical biography. It is mortifying to reflect that even the names of those who have rendered signal service to humanity by discoveries or improvements in medical science, are barely known to the intelligent portion of the public. It would probably be difficult to find many persons in any community, out of the ranks of the medical profession, who are aware that such a man as Laennec ever lived! Or, to take a more striking illustration, how small a proportion of these, who would blush to be considered wanting in general information, are familiar with the name of the immortal discoverer of vaccination ; and yet, owing to the genius, boldness, industry and perseverance of Jenner, millions have been saved from death by the most loathsome of diseases! I cannot but think that the public estimation of our profession would be in no small measure enhanced, were a fair share of the popular biographical literature allotted to the lives and labors of medical men.

To the student in medicine, medical biography is especially to be commended, not merely as an attractive province of polite learning, but because it cannot fail to exert a salutary and lasting influence upon the character. Let the young mind anxious to fulfill its mission, go to the past and study the lives and labors of the great and good men, whose destinies in this world have been accomplished. Who can doubt that it is in the order of Providence for the illustrious dead to serve as shining lights for the living! To illumine the paths of those who are to follow in their footsteps, was a part of their mission. This thought should stimulate the desire to live beyond this bodily existence, in the cherished remembrance of mankind. It is a noble and virtuous ambition to strive thus to survive on earth the brief term of mortal life!

The career of the distinguished man whose biography has been our theme on this occasion, is pre-eminently worthy of admiration. In his character were beautifully blended the finest intellectual and moral qualities of our nature. With mental powers of the highest order, were combined simplicity, modesty, purity and disinterestedness, in such measure that we feel he was a man to be loved not less than admired. His zeal and industry in scientific pursuits were based on the love of truth for its own sake, and a desire to be useful to his fellow men. To these motives to exertion much of his success is to be attributed. Mere intellectual ability and acquirements do not qualify either to make or to appreciate important scientific discoveries. The mind must rise above the obstructions of self-love, jealousy and selfish aims. Hence it is, that most of those who have attained to true eminence in the various paths of scientic research, have been distinguished for excellencies of the heart as well as of the head.

The example of Laennec is worthy of our imitation. His superior natural gifts we can only admire. His mission was far above aught that we can hope for. But we can imitate the industry, without which, his genius would have been fruitless. We may aspire to emulate his virtues. It is neither arrogance nor folly to be inspired by ambition in contemplating intellectual and moral attainments which are beyond our reach. We are commanded ever to strive for Divine perfection. Let us show our reverence for the memory of Laennec by endeavoring to follow humbly in his footsteps.—*New Orleans Medical News and Hospital Gazette.*

